# Cell Cycle Model System for Advancing Cancer Biomarker Research

**DOI:** 10.1038/s41598-017-17845-6

**Published:** 2017-12-21

**Authors:** Iulia M. Lazar, Ina Hoeschele, Juliana de Morais, Milagros J. Tenga

**Affiliations:** 1 Department of Biological Sciences, Virginia Tech. 1981 Kraft Drive, Blacksburg, VA 24061 USA; 2 Carilion School of Medicine, Virginia Tech 1981 Kraft Drive, Blacksburg, VA 24061 USA; 3 Department of Statistics and Biocomplexity Institute, Virginia Tech 1015e Science Circle, Blacksburg, VA 24061 USA

## Abstract

Progress in understanding the complexity of a devastating disease such as cancer has underscored the need for developing comprehensive panels of molecular markers for early disease detection and precision medicine applications. The present study was conducted to assess whether a cohesive biological context can be assigned to protein markers derived from public data mining, and whether mass spectrometry can be utilized to screen for the co-expression of functionally related biomarkers to be recommended for further exploration in clinical context. Cell cycle arrest/release experiments of MCF7/SKBR3 breast cancer and MCF10 non-tumorigenic cells were used as a surrogate to support the production of proteins relevant to aberrant cell proliferation. Information downloaded from the scientific public domain was queried with bioinformatics tools to generate an initial list of 1038 cancer-associated proteins. Mass spectrometric analysis of cell extracts identified 352 proteins that could be matched to the public list. Differential expression, enrichment, and protein-protein interaction analysis of the proteomic data revealed several functionally-related clusters of relevance to cancer. The results demonstrate that public data derived from independent experiments can be used to inform biological research and support the development of molecular assays for probing the characteristics of a disease.

## Introduction

The discovery of biomarker panels of high sensitivity and specificity is pursued at every level of diagnostics, from preliminary screening for the presence or risk of a disease, to staging, response to treatment, progression or relapse. Biomarker potential has been associated not only with the biological presence of various biochemical components (nucleic acids, proteins, carbohydrates, lipids or small molecules), but also with their cellular location and change in expression level or chemical modifications (mutation, epigenetic or PTMs)^1–6^. Despite all efforts, however, no biomarker profiling effort has led yet to a satisfactory panel that enables sensitive and specific detection of relevant molecular markers in specific tissues or body fluids. On the other hand, the advance of high-throughput sequencing and mass spectrometry (MS) technologies resulted in the generation of massive amounts of data that can provide researchers with previously inaccessible insights into the functionality of a biological system^7^. Disease-relevant information emerging from comprehensive datasets stemming from whole-genome expression, transcriptome, proteome or other omics profiles is produced at ever increasing rates and compiled in data repositories. For example, one of the first gene panels derived from microarray experiments is the 70 gene signature (70-GS), so-called MammaPrint^TM^ assay, that was developed for breast cancer diagnostics and prognostics intended for individualized treatment of estrogen receptor (ER)+/−, lymph-node (−) patients^8^. An expression pattern of 534 “intrinsic” genes was used for breast cancer classification^9^, and additional prognostic profiles such as the 76-gene assay Rotterdam Signature, the 21-gene recurrence score Oncotype Dx®, the PAM50 Risk of Recurrence score, the EndoPredict®, and the Breast Cancer Index, were developed^10,11^. Nonetheless, the cost of generating large biological datasets that would enable the development of such biomarker panels and translating the findings into medical practice is not trivial. Such challenges suggest that discovery efforts should be revisited to better capitalize not just on novel technological advancements, but also on the availability of the vast amount of already existing data.

Our work on proteomic profiling the G1 cell cycle stage of MCF7 breast cancer cells has led to the conclusion that biomarker proteins are not isolated players in the disease but rather part of highly interconnected functional networks^12,13^. Three broad protein-protein interaction (PPI) networks were recognized: signaling, DNA damage repair, and metabolism/oxidative stress. Capitalizing on information extracted from the scientific literature and public databases, the focus of this work was to investigate whether: (a) functionally-related gene or protein categories can be extracted from the totality of markers catalogued in various data repositories; (b) cell cycle experiments and MS can enable protein-level detection of such categories in multiple cell lines and cell states; (c) PPI networks can expose new relationships between the marker proteins; and (d) protein clusters of relevance show propensity for detection in tissues or blood to support the development of minimally invasive diagnostic assays.

## Results

### Detectability of cancer-associated proteins in cell lines

**Figure 1:**
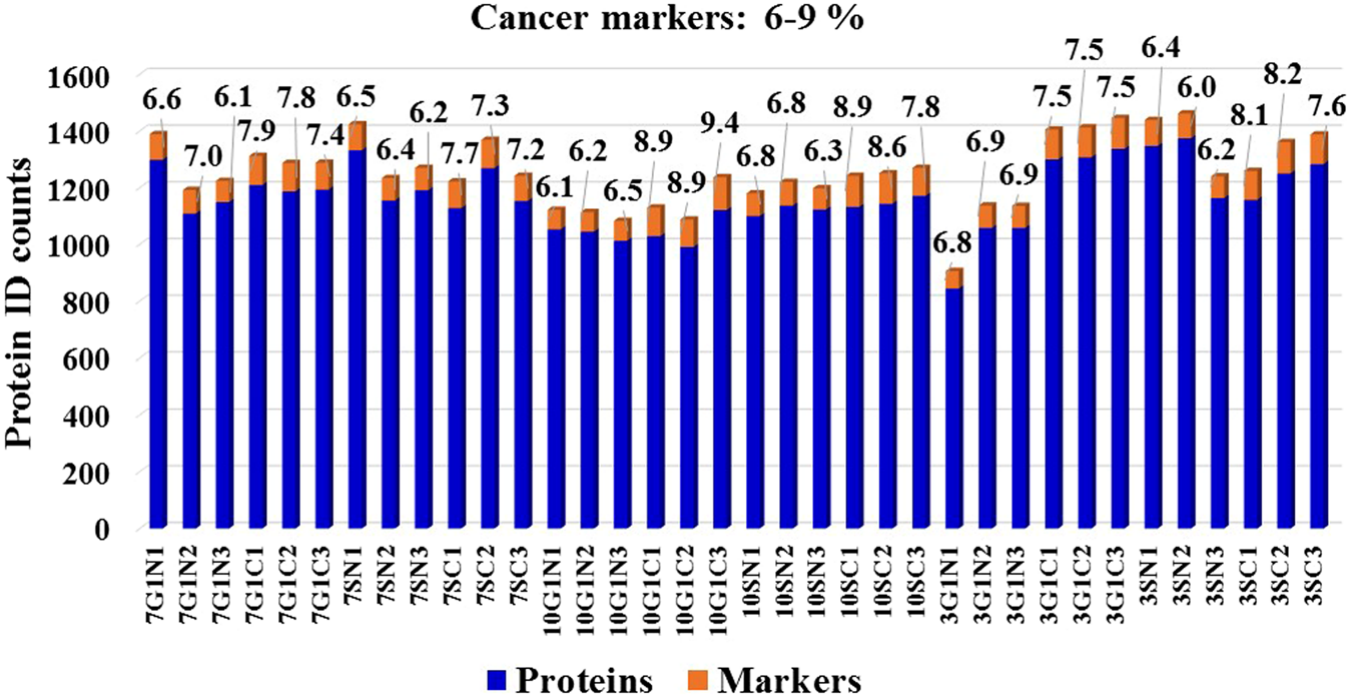
Bar graph displaying the % cancer-associated proteins identified in each cell state of MCF7, MCF10 and SKBR3 cells. The protein ID counts represent the number of proteins identified in five combined technical replicates of each cell state. Biological replicates are numbered as 1, 2 and 3.

**Figure 2:**
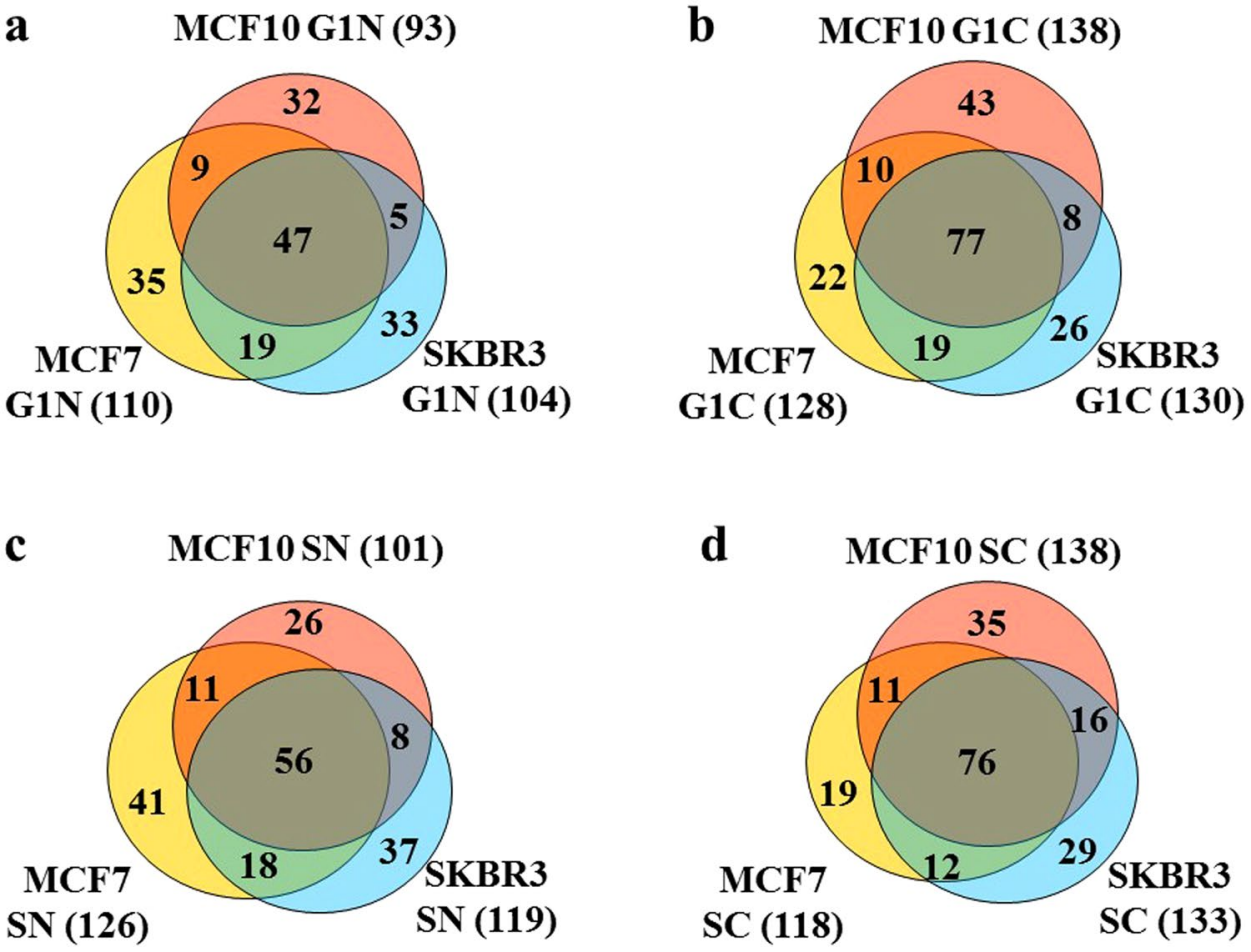
Venn diagrams displaying the overlap in cancer marker protein identifications in different cell states (G1N, G1C, SN, and SC) between MCF7, MCF10 and SKBR3 cells. The diagrams were built from combined marker identifications in three biological replicates. The total number of markers per cell state is provided in parenthesis. (**a**) G1N: unique markers 180; (**b**) G1C: unique markers 205; (**c**) SN: unique markers 197; (**d**) SC: unique markers 198.

**Figure 3:**
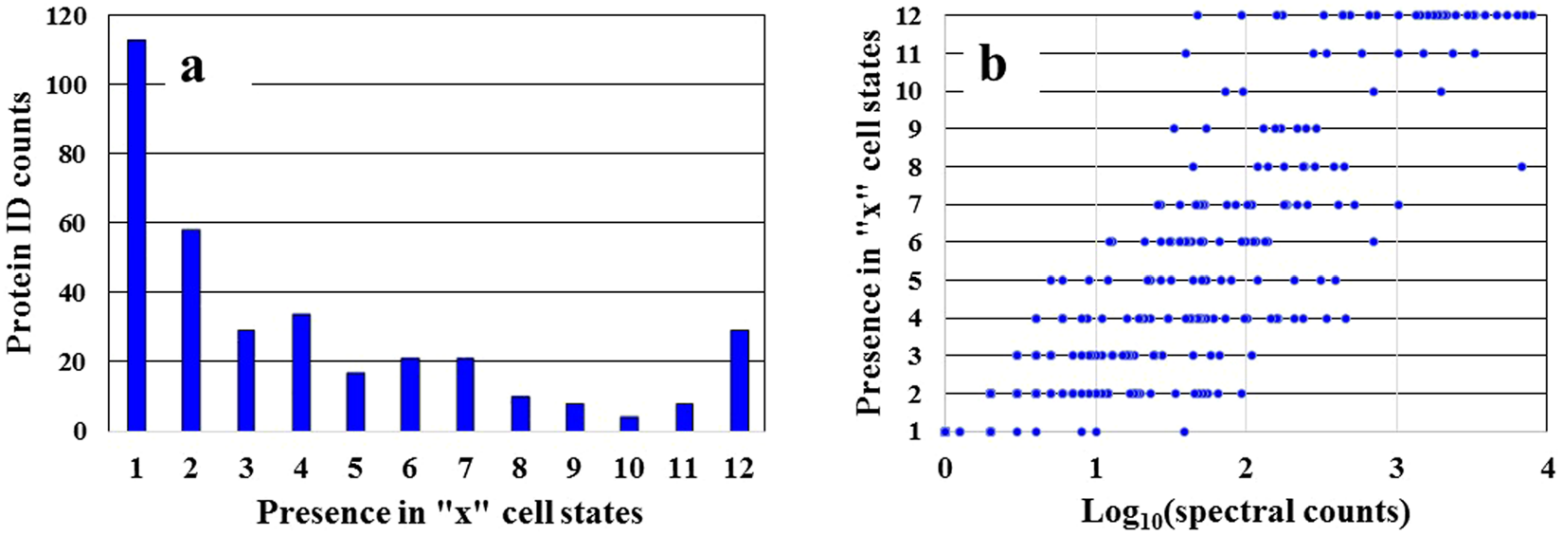
Charts displaying the detectability of cancer marker proteins in any number “x” of cell states. (**a**) Number of cancer marker proteins that could be identified in “x” cell states. (**b**) Detectability of cancer marker proteins in “x” cell states as a function of the log-transformed number of matching spectral counts (x = 1–12 cell states corresponding to the G1/S cell cycle stages and nuclear/cytoplasmic fractions generated from the MCF7, MCF10 and SKBR3 cells).

To maximize protein coverage and the identification of proteins with biomarker utility, proteome profiles of representative breast cancer [MCF7/ER+ and SKBR3/HER2+] and non-tumorigenic (MCF10A) cell lines were generated for different stages of the cell cycle by nano-liquid chromatography (LC)-MS. The cells were cultured in optimized growth medium, arrested in G1 by serum deprivation, released in S in the presence of growth factors and/or hormones, and separated into enriched nuclear (N) and cytoplasmic (C) fractions. The corresponding cell states were termed G1N, G1C, SN and SC (12 cell states for 3 cell lines). Proteomic analysis of three biological replicates of each of these cell states led to the identification of 906–1462 proteins matched by ~3050–4670 spectral counts per cell state, with an intra-state coefficient of variation (CV) of 1–12%. The FDR for peptide identifications was <3% (Fig. 1). Cancer-relevant literature^1–6^ and the UniProt *Homo sapiens* database^14^, encompassing 20,198 reviewed unique protein sequences, were mined with bioinformatics tools enabled by the DAVID resource (Database for Annotation, Visualization and Integrated Discovery)^15,16^ to identify the proteins associated with cancer development. The search resulted in the compilation of a list of 1038 proteins from the entire human proteome, of which, 352 were identifiable in the cell cycle experiments. From here on, we will refer to these proteins as cancer markers. A number of 62–116 putative markers were identified per cell state, representing 6.0–9.4% of the total protein IDs (Fig. 1). Supplemental Table S1 provides the list of 1038 proteins, as well as the 352 proteins identified in the cell cycle experiments with their corresponding UniProt IDs, total spectral counts accumulated from all cell states, mean spectral counts calculated from three biological replicates, and the associated CVs. The overlap between the identifiable proteins in the G1N/G1C/SN/SC cell cycle states is shown in Fig. 2. While a large proportion of proteins (~33%) were identifiable only in a single cell state (Fig. 3a), detectability in multiple states improved with the number of spectral counts per protein (Fig. 3b), indicating that the somewhat low level of overlap (25–38%) in the Venn diagrams from Fig. 2 was rather due to less than optimal detectability of low abundance proteins than differences in expression. The analysis of similar cell fractions on more sensitive MS platforms is expected to increase not only the number of protein IDs, but also the overlap and reproducibility of their identification.

### Up/down-regulated protein panels

**Figure 4:**
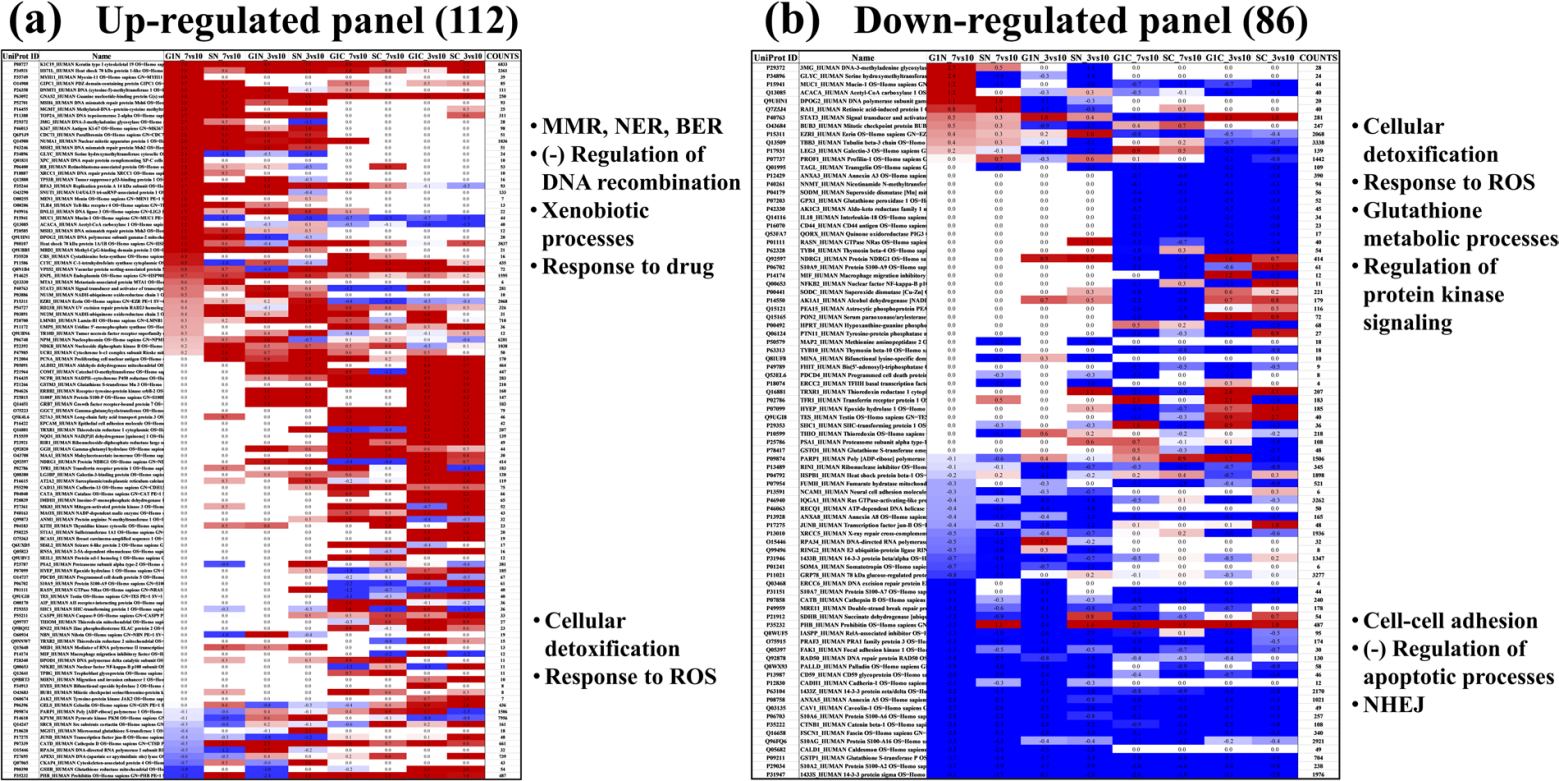
Heat maps of cancer marker proteins that changed spectral counts in the cell cycle arrest/release experiments. (**a**) Up-regulated panel of 112 proteins (red); (**b**) Down-regulated panel of 86 proteins (blue). Protein descriptors are provided in columns to the left (UniProt IDs and name), and total number of spectral counts to the right, of each heat map. Major biological processes affected by a change are indicated in the upper and lower levels of each panel.

To explore the space of proteins that could potentially reveal differences between cancerous and non-cancerous cell states, the MCF7 and SKBR3 cells were compared to MCF10 in their respective cell cycle stages and cellular fractions. The differences were assessed based on MS spectral count data, and the results were compiled in two panels, displaying up- or down-regulation, respectively. In total, 8 comparisons of cancerous to non-cancerous cell states were made, the conditions for inclusion being: (i) the proteins had to be identifiable in at least two of any 12 cell states; (ii) the fold-change (FC) in spectral counts of the MCF7 or SKBR3 to MCF10 comparisons had to be approximately larger than two [more precisely, log_2_(FC) >0.95 or log_2_(FC) <(−0.95)]; and (iii) the individual p-values associated with a change had to be <0.2 for at least one out of the 8 comparisons. A few comments must be made in regard to this selection process. First, due to poor reproducibility in the detection of low-abundance proteins, we opted for a relaxed p-value for protein inclusion in the two panels to better capture the trends of the global cellular processes that may change in response to a perturbation. Second, the goal was not to produce differential expression lists characteristic to our particular arrest/release experiments, controlled at a certain significance level. The protein list under discussion was pre-selected based on independent criteria (in this case by data mining), and comparisons were performed to assess whether any of the proteins could qualify as promising targets for cell cycle experiments due to potential changes in expression level. Therefore, correction for multiple testing was not performed, and the p-values should be viewed solely as a cut-off filter for eliminating proteins with irreproducible detection, and not be expected to control the overall false positive rate. The log_2_ values of the fold-change with the associated statistical parameters are listed in Supplemental Table S1. Based on the above selection criteria, a total of 177 proteins (83% with p ≤ 0.05, 4.5% with 0.05 <p ≤ 0.1, and 12.5% with 0.1 <p ≤ 0.2) identified by ≥4 spectral counts qualified for inclusion in the two panels, of which 112 were associated with up- and 86 with down-regulated states, respectively. Supplemental Table S2 encompasses the two panels, cells comprising zeros corresponding to proteins that were not detected in that given cell state. The column on the far right represents the sum of all spectral counts that matched a particular protein ID. Figure 4a and b depict the heat maps of the 112 and 86 proteins. The columns were arranged in order of cell cycle stage and compartment (G1N7, SN7, G1N3, SN3, G1C7, SC7, G1C3, SC3), and the proteins were sorted based on the values in the first column that represent the log_2_ ratios of the G1N/MCF7 vs. MCF10 comparison, from high to low. While some proteins (21 in number) displayed both up- and down-regulation in different cell states, clear trends were observable in both nuclear and cytoplasmic fractions, extreme changes being characteristic rather to the nuclear fractions. We found that for our cell cycle experiments, by sorting the data based on changes in a cell state of reference (in this case the G1 nuclear fraction of MCF7 vs. MCF10), the biological processes that were affected by the perturbations associated with arrest/release lined up naturally in an easy to interpret manner. Having the nuclear fractions separated from the cytoplasmic ones helped not only increasing the protein coverage but also localizing the sources of the change. The results were validated with endogenous β-actin and β-tubulin, and spiked bovine standards (hemoglobin, α- and β-casein). In addition, two protein barcodes developed in our laboratory for the validation of spectral count data for nuclear (11 proteins) and cytoplasmic (62 proteins) cell fractions generated in cell cycle experiments^17^ were tested. All endogenous, spike and nuclear barcode proteins displayed <2-fold change in spectral counts. A few cytoplasmic barcode proteins showed a slightly higher than 2-fold change, but only in a few cell fractions. This was, however, an expected outcome, as the barcode was developed from MCF7 and MCF10 datasets only.

### Annotation, enrichment and analysis of protein-protein interactions

**Figure 5:**
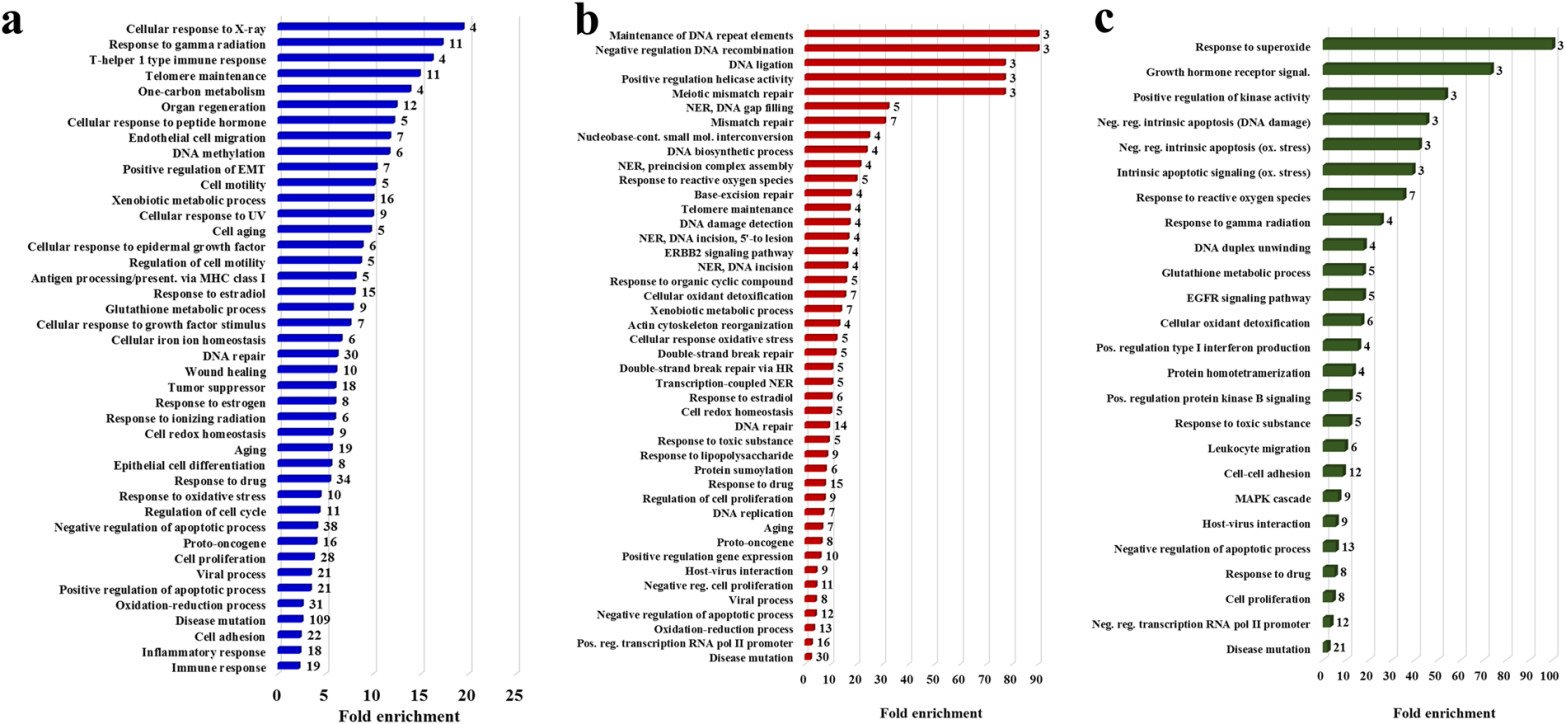
Bar graphs displaying the enriched GO biological processes associated with the cancer marker proteins. (**a**) Full list of 352 cancer markers identified in cell cycle experiments; (**b**) List of 112 up-regulated proteins; and (**c**) List of 86 down-regulated proteins. Only categories with >2-fold enrichment and FDR <6% are shown. Labels on the right of each bar indicate the number of proteins that matched that particular enriched category. Enrichment status was assessed with DAVID tools.

**Figure 6:**
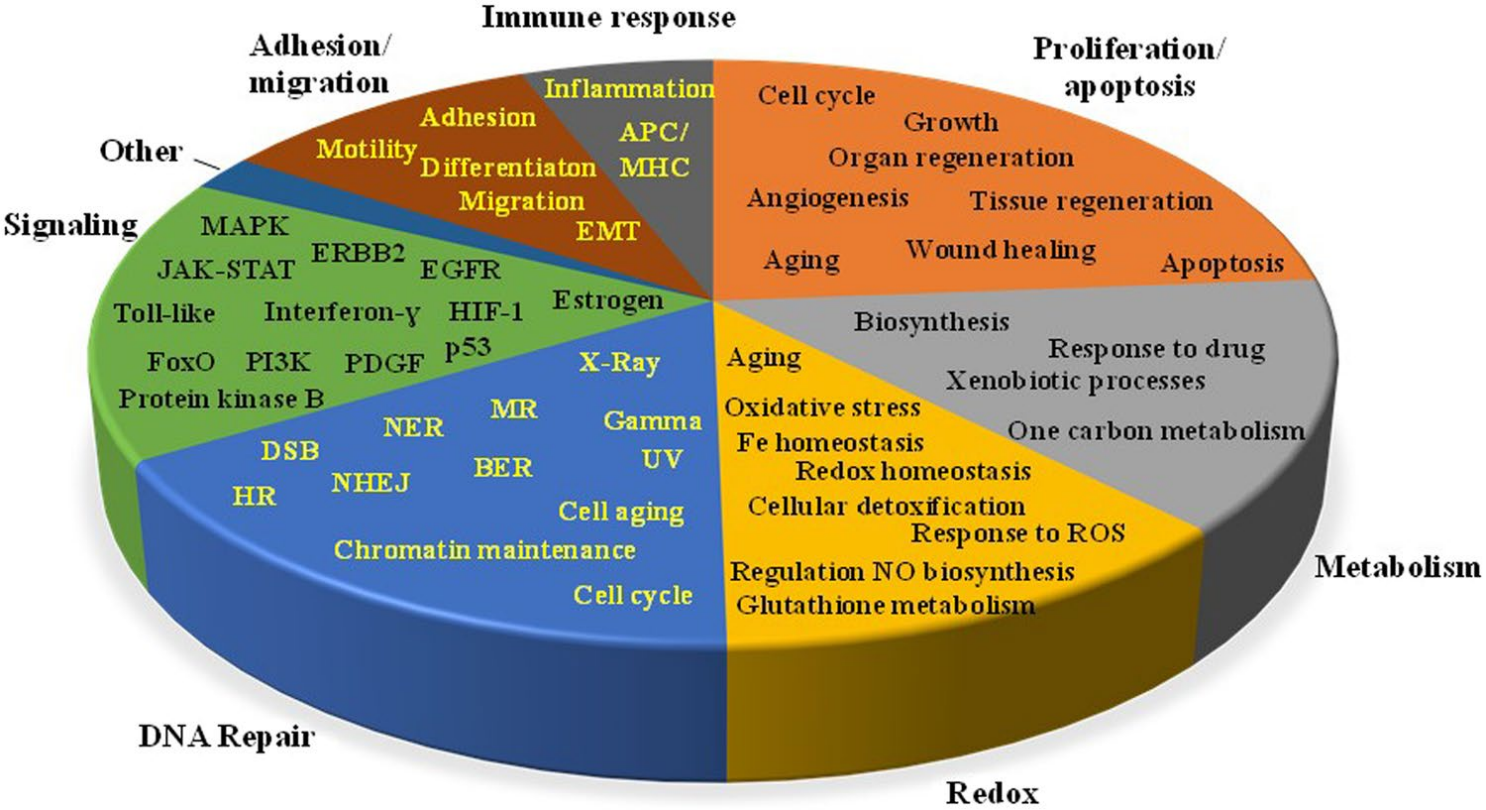
Pie chart summarizing the most relevant biological processes associated with the cancer marker proteins, grouped in seven major categories: signaling, DNA repair, redox processes, metabolism, proliferation/apoptosis, immune response, and adhesion/migration.

**Figure 7:**
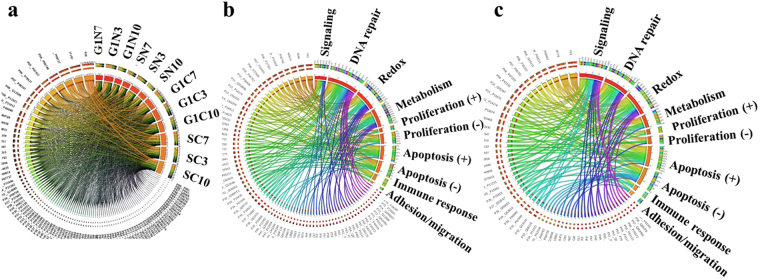
Circos plots displaying protein associations with cell cycle and up/down-regulated biological processes. (**a**) Protein associations with cell cycle stages and cell fractions; to avoid overcrowding, only the top 100 most abundant proteins are listed (out of 352), and line width is reflective of the spectral counts per protein; (**b**) Protein associations of up-regulated proteins with the major biological categories; (**c**) Protein associations of down-regulated proteins with the major biological categories. The protein IDs are listed in all plots in decreasing order of protein hits per category, counterclockwise.

**Figure 8:**
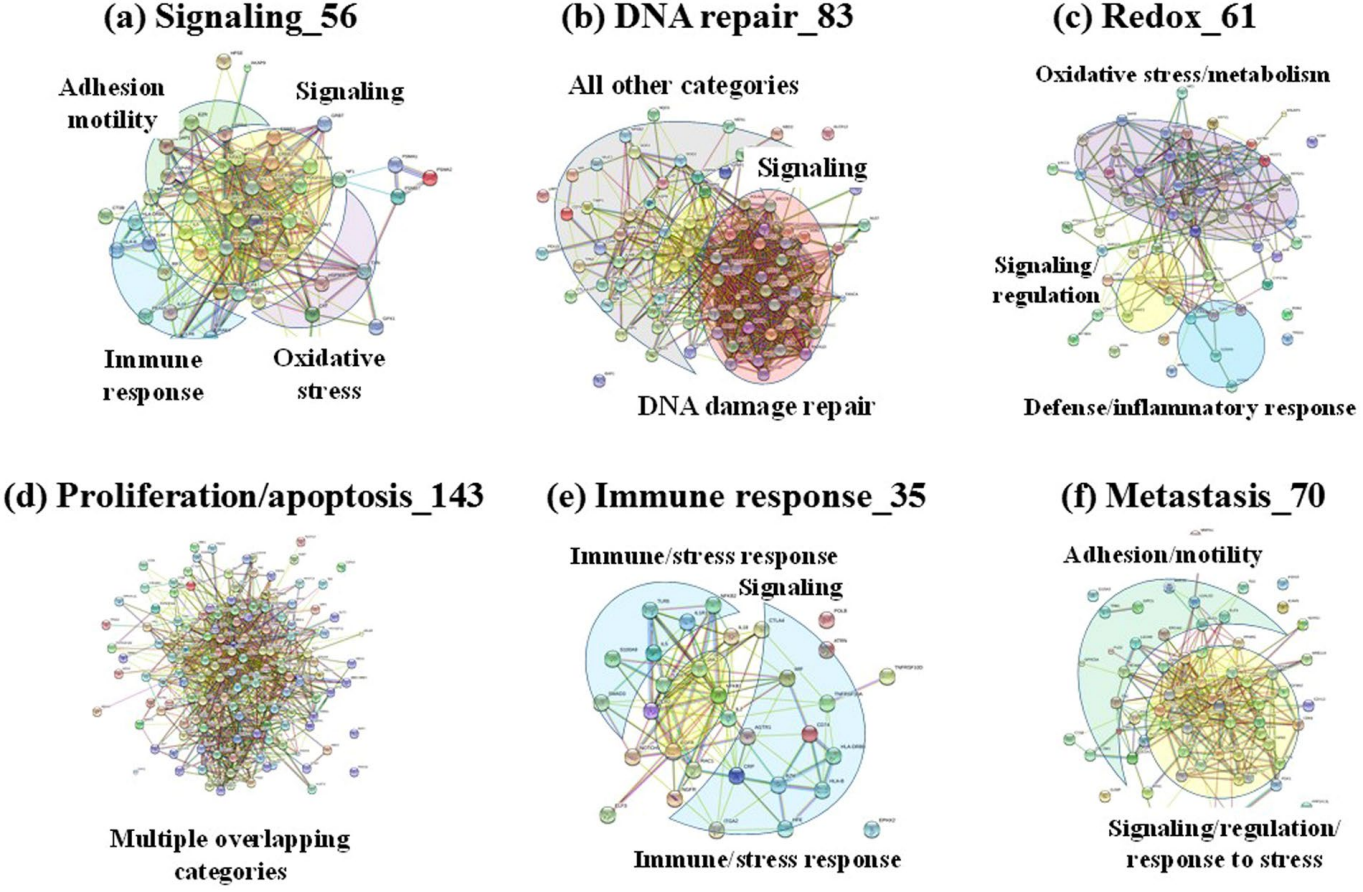
STRING PPI networks of major biological categories. The total number of proteins included in each network is shown, and the major protein interaction hubs are color-coded in each figure.

To place the experimental findings in biological context, the protein sets identified in the cell cycle studies were annotated and assessed for enrichment in GO (gene ontology) categories^18^. Supplemental Table S3 comprises the results for the lists of 352, 112-up and 86-down regulated proteins for the following categories: biological process, molecular function, cellular compartment, Kegg pathways, associated diseases and presence in tissues. Only the records for which the FDRs were <6% are listed. Relevant biological processes for the list of 352 and differentially expressed proteins are presented as bar graphs in Figs 5 and 6 as a function of fold-enrichment, or, shown categorized in a pie chart. Specific protein associations with various cell cycle stages and up/down-regulated biological categories were captured in Circos plots^19^ (Fig. 7 and Supplemental Table S4), and PPIs were visualized with STRING (Search Tool for the Retrieval of Interacting Genes^20^) (Fig. 8). PPIs specific to cell lines, cell-cycle stage, and differential expression are provided in Supplemental Fig. S1.

### Diagnostics/propensity for detection in tissues and blood

**Figure 9:**
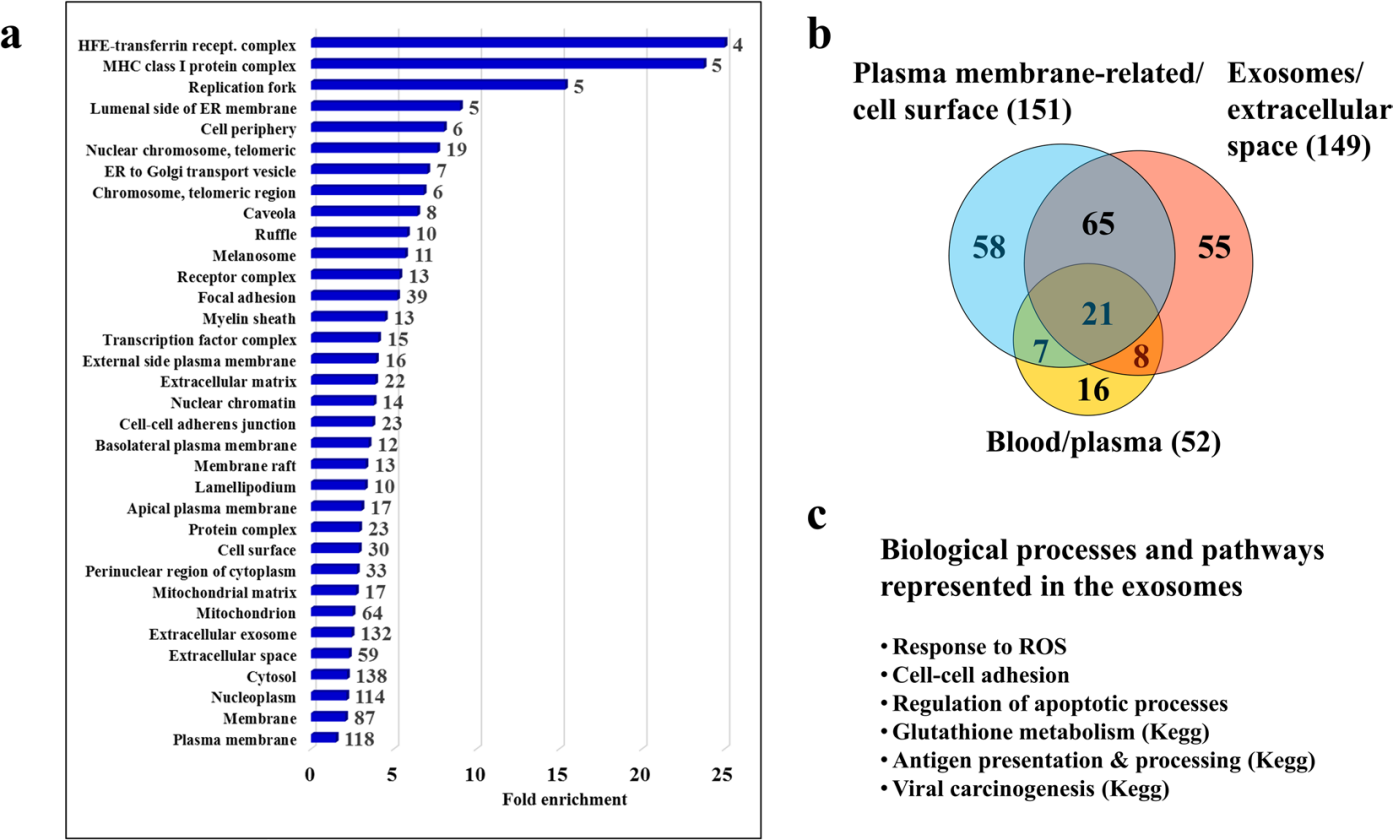
Detection propensity for the list of 352 cancer marker proteins. (**a**) Bar graph displaying enriched GO cellular compartments. Only categories with >2-fold enrichment and FDR <6% are shown. Labels on the right of each bar indicate the number of proteins that matched that particular category. Enrichment status was assessed with DAVID tools. (**b**) Venn diagram displaying the overlap between cancer marker proteins associated with the plasma membrane/cell surface, the exosome/extracellular space, and the proteins that have been previously identified in blood or plasma. (**c**) Biological processes and pathways represented by exosome proteins.

To further assess whether the analysis of cell lines can lead to the identification of protein panels with diagnostic potential, the cellular location and prior identification of these proteins in tissues was explored. Supplemental Table S3 provides a list of enriched GO categories in terms of cellular location and tissue associations for the list of 352 proteins. Figure 9 captures the categories with larger than 2-fold enrichment, as well as the overlaps between proteins associated with the plasma membrane/cell surface, the ones identified in blood or plasma, and the proteins pertaining to the extracellular space and a rather new source of biomarkers, i.e., exosomes.

## Discussion

In recent studies, we identified three major functional categories of cancer markers in the G1 stage of the MCF7 breast cancer cell cycle, i.e., signaling, DNA repair, and oxidative stress^12,13^. In the present work, we expanded our analysis to multiple cell lines, and refined the categories based on results obtained by mining the literature and the *Homo sapiens* protein database, effort that resulted in the identification of 1038 proteins associated with the development of cancer. We reasoned that a large list, in excess of 1000 proteins, generated by world-wide independent research experiments would be able to reveal a comprehensive view of the altered protein landscape characteristic to cancerous cell states. From this list, 352 proteins were identifiable in G1-arrest/S-release cell cycle experiments of MCF7/ER+, SKBR3/HER2+ and MCF10 non-tumorigenic cells. The list of 352 proteins proved to be a fair representation of the compiled list of 1038 in terms of GO categories of biological processes, pathways, and cellular location. Therefore, we suggest that cell cycle experiments conducted with relevant cell lines, under appropriate biological perturbations, could be used as a model system for simultaneously assessing the behavior of a large set of proteins with biomarker potential and for advancing hypotheses that can inform future clinical research.

We evaluated the ability to detect the presence and change in expression of protein markers of interest in the context of their biological relevance to cancer. Each cell state (G1N, G1C, SN and SC) enabled the identification of a substantial number of markers (Fig. 1). While differences between cell lines could not be assessed solely based on protein IDs, due to the variability introduced by the low abundance proteins (Figs 2 and 3), 29 out of 352 proteins were found to be common to all cell lines and cell states. Moreover, as noted in the Circos plot from Fig. 7a, the distribution of high and low-abundance markers throughout various cell lines and cell cycle stages was rather uniform, with the cytoplasmic fractions comprising a somewhat larger number of marker proteins than the nuclear ones. Most proteins were associated with multiple cell fractions and cell cycle stages. The majority of top-enriched biological processes that emerged from the list (Fig. 5a) could be grouped into a few relevant categories, representative of a molecular-to-cellular trail of cancer progression: signaling, DNA damage repair, oxidative stress and metabolism, (+/−) regulation of cell proliferation and apoptosis, immune response, and adhesion/migration/differentiation (Fig. 6). Disease mutation proteins were abundant in the list (107 out of 352), and included components of all major biological categories. Specialized processes were generally represented by a smaller number of proteins with more pronounced enrichment (>10–25-fold), while broader and more inclusive categories were represented by a larger number of proteins with a smaller fold-enrichment (<10-fold). In the followings, we will focus the discussion on the most distinct trends that evolved from analyzing the data.

Figure 6 encompasses the most relevant biological processes, represented by the cell cycle markers, grouped in 7 major categories. Several proteins were common to multiple processes within the same category. Cellular signaling was represented by pathways that drive proliferation in ER+ and ERBB2+ cancer cells (e.g., MAPK, EGFR/ERBB2 and estrogen signaling), and additional downstream pathways involved in proliferation, apoptosis and survival. Components of the PI3K-PKB-FOXO signaling axis, with roles in aging and lifespan, were present, as well^21^. Pathways such as p53, PDGF, interferon-ɣ, Toll-like and JAK-STAT further correlated the above pathways to DNA damage repair, angiogenesis, migration and immune response. Survival pathways such as HIF-1, activated by cancer cells in response to hypoxic stress, had components that have been recognized recently as novel targets for cancer therapies^22^. Multiple constituents of the signaling cluster, in particular the cell surface receptors, the kinases and the activators of immune response, are well-known proto-oncogenes that present special interest as cancer biomarkers or drug targets.

The DNA damage repair category encompassed the entire range or repair mechanisms^23^, as well as chromatin maintenance, response to radiation and cell aging. Dysfunctional DNA damage repair pathways that lead to the accumulation of mutations and genomic instability have been the target of many drug discovery and development efforts^24,25^. Multiple damage repair proteins overlapped the categories of cell cycle/proliferation and apoptosis, which, in turn, contained both positive and negative regulators. Positive regulation of apoptosis correlated with negative regulation of cell proliferation through proteins such as programmed cell death (PDCD5), BCL-2 homologous antagonist killer, and NOTCH1-a receptor for membrane bound ligands that is implicated in cell-fate determination through processes that relate not just to cell proliferation and apoptosis but also differentiation, migration and angiogenesis. Alternatively, negative regulation of apoptosis correlated to proliferation in general, or to positive regulation of proliferation. This was supported by a large number of proteins with complex roles in chaperoning, histone assembly, centrosome duplication and regulation of tumor suppressor p53 (NPM), sensors of DNA damage repair via non-homologous end joining (NHEJ) and double strand break (DSB) mechanisms (PRKDC and MRE11A), cell-cell adhesion and differentiation (EPCAM), transcription of RNA polymerase II-dependent genes (MED1), innate immune response (MIF), and signaling via the epidermal growth factor receptor (EGFR). Additional proteins involved in negative regulation of apoptosis, such as STAT3 that mediates cellular responses to growth factors and interleukins, and the tumor suppressor phosphatase PTEN that antagonizes the PI3K-AKT/PKB signaling pathway and cell survival, had roles in both positive and negative regulation of proliferation. Angiogenic pathways, as a whole, did not qualify for the list of enriched biological categories, but relevant proteins were present and common to pathways related to proliferation, invasion/migration and immune response.

Redox processes emerged as a particularly relevant sub-category of metabolism. Oxidative stress was intertwined with glutathione metabolism, aging and NO biosynthesis, and included a range of protein functional categories involved in antioxidant defense, cellular detoxification and apoptotic signaling (e.g., reductases, transferases, peroxidases, and antioxidants)^26–31^. Deregulation of NO production and transport has been associated with endothelial dysfunction and angiogenesis-related disorders^32^. Processes related to iron metabolism and homeostasis in cancer have been implicated in the production of reactive oxygen species (ROS), cell respiration and detoxification, as well as cell cycle, growth and proliferation^33^. Among many biosynthetic metabolic pathways, one carbon metabolism, a pathway that lies at the basis of DNA/RNA synthesis and DNA methylation processes, has emerged in recent years as an important area of study, mainly due to implications in diseases such as cancer, aging, loss of cognitive function, stroke and cardiovascular diseases^34^. Xenobiotic and response to drug processes represented another category of interest, especially if assessed in the context of future personalized medicine applications^35,36^.

Cancer immunotherapy is not a new concept, however, it is only recently that antibody and cell-based therapies have been effectively implemented. Both innate and adaptive immune responses, involving the activation of effector molecules, pathways and cells, have been shown to co-operate in killing the cancer cells^37,38^. The proteins that could be mapped to the immune response category were part of various defense, stress and inflammatory response mechanisms, antigen presentation, and cytokine and chemokine production. Notably, overlaps with multiple signaling pathways (e.g., MAPK, Toll-like, JAK-STAT, NF-Kβ, metabolic ROS regulation) were observable.

Proteins associated with the level of cancer cell differentiation, cell adhesion, motility and migration have been recognized to have diagnostic value due to their relevance to metastatic processes^39,40^. Epithelial to mesenchymal transition (EMT) followed by loss of adhesion proteins (e.g., cadherins), degradation of the extracellular matrix (ECM) by matrix metalloproteinases (MMPs), cathepsins and the urokinase-type plasminogen activator (uPA) system, as well as co-option of factors from the tumor microenvironment to support metastasis (e.g., cytokines TGF-β and SDF1), are all processes that provide cancer cells with the ability to invade blood vessels and migrate to distant sites in the body. Existing data underscore the complexity of the metastatic process and the divergent evolution of cancer cells at the metastatic site, pinpointing that successful therapies will need to target not just the cancer cells alone, but also the tumor microenvironment.

Global PPI networks indicated a high level of interconnectivity within and between the selected protein categories, the three major groups (signaling, DNA damage repair, and oxidative stress/metabolism) being abundantly present in all cell lines (Supplemental Fig. S1a–c). These groups were also identifiable in all four cell cycle stages (Supplemental Fig. S1d–g), with DNA damage repair and signaling being dominant in the nuclear cell fractions (Supplemental Fig. S1d and f). Refined networks, based on enriched biological processes, revealed intimate interactions that superimposed the selected category with a number of other categories, suggesting that a meaningful evaluation of such processes should not be performed in isolation (Fig. 8). Essentially, all marker categories were present at some extent in all interaction networks, with regulatory signaling transcending all clusters. From the total of 352 proteins, 244 were part of the networks shown in Fig. 8, shared proteins between multiple networks being associated with regulation of signal transduction, apoptotic processes, response to stress and immune response. The composition, structure and dynamics of PPI networks have been shown to be perturbed in diseased biological states, and, therefore, such networks have been recognized as valuable tools for exploring the molecular foundations of disease^41,42^. Moreover, targeting PPI networks instead of single proteins has emerged as a new therapeutic paradigm for systemic diseases such as cancer.

The quantitative comparisons between the cancerous (MCF7, SKBR3) and non-cancerous (MCF10) cell lines were reflective of the enriched biological categories highlighted above (Fig. 5b and c). A breakdown of the data in heat maps, based on cell cycle stage and compartment, revealed more specifically the source of the observed changes (Fig. 4). The nuclear fractions showed the most extreme variations. Various components of the DNA repair machinery were up-regulated in both G1N and SN cancerous cell states. Mismatch repair (MMR), nucleotide excision repair (NER), base excision repair (BER), homologous recombination (HR) and negative regulation of DNA recombination were among the top most-enriched and up-regulated biological processes (Fig. 4a/top). Up-regulated metabolic xenobiotic/drug response proteins were present in both nuclear and cytoplasmic fractions, and mitochondrial-related responses to ROS/cellular detoxification and redox homeostasis showed elevated expression in the cytoplasmic fractions, in particular in SKBR3 cells (Fig. 4a/bottom). However, regulation of oxidative stress-induced death, cellular detoxification via superoxide dismutase activity, along with glutathione metabolism also displayed down-regulated trends in the cytoplasmic fractions (Fig. 4b/top). Cell-cell adhesion and negative regulation of apoptosis were among the most down-regulated processes in both nuclear and cytoplasmic fractions (Fig. 4b/bottom). In addition, the NHEJ and cancer proteoglycans pathways were identified as down-regulated in the panel. As several proteins involved in cell-cell adhesion and cytoskeletal reorganization are also part of the proteoglycan family, it is worth noting that heavy glycosylation or changes in the structure of glycosylation may affect the detection ability of proteins and alter the interpretation of results. In such cases, the observed alterations are not necessarily the result of changes in expression level, but of changes in the nature, site, number, or structure of PTMs. Likewise, protein translocation from one cellular compartment to another can also affect the representation of differential expression data.

The bearing of diverse, complex and interconnected pathways that regulate the multiple facets of the same biological processes cannot be overstated when one considers systemic changes in a complex environment such as the cell. The STRING PPI diagrams (Supplemental Fig. S1h and i) and the Circos plots from Fig. 7b and c that encompass the differentially expressed panels, expose the complex, multifunctional role played by most marker proteins. To avoid overcrowding, only the proteins that were included in the seven categories were incorporated in the Circos plots (77 and 60, respectively). The proliferation/apoptosis category was broken down in four sub-categories to better capture the impact of (+) and (−) regulatory components. With only 21 overlapping proteins between the two panels, the independent and opposing forces that act on each category are evident. Overall, however, the emerging trends from Figs 6 and 7 and Supplemental Fig. S1 are indicative of faulty DNA damage repair mechanisms that lead to accumulation of mutations, genomic instability, and resistance to DNA-damaging therapies. Such altered mechanisms, backed by deregulated responses to oxidative stress, support cancer cells in their quest for survival^43^. BER and NER are particularly important for removing lesions caused by ROS and UV/ionizing radiation, respectively, throughout all cell cycle stages, while MMR plays an important role mainly in S, where it corrects for base-base mismatches and small insertions and deletions. Double strand breaks are typically repaired by NHEJ in G1 and homologous recombination (HR) in S and G2^44^. An altered, non-functional NHEJ repair machinery in G1 can be replaced by an alternative mechanism, microhomology mediated end joining (MMEJ)^45^. However, this mechanism is more error-prone than NHEJ. In human cell lines, proteins such as DNA ligase 1/3, PARP and histone H1 have been shown to be involved in the MMEJ mechanism. A few examples of high and/or consistent up-regulation included proteins associated with DNA replication (RPA3, TOP2A, NUMA1, DNMT1), DNA damage repair (MSH proteins, TP53 binding proteins, LIG3), proliferation (KI67, PCNA, PHB), vesicle mediated sorting (VPS52), and adhesion (epithelial cell adhesion and tumor-associated calcium signal transducer 1 EPCAM antigen). Prevalent up-regulation in MCF7/ER+ cells was observed for MMR proteins, signal-transducer GNAS, intracellular protein breakdown CTSD, and the epithelial marker cytokeratin 19. Specific to SKBR3/HER2+ cells, on the other hand, were the receptor tyrosine kinase ERBB2, growth factor receptor bound protein (GRB7), breast carcinoma amplified sequence (BCAS1), S100 calcium binding protein P (S100P), aldehyde dehydrogenase 2 (ALDH2), and catechol-O-methyltransferase (COMT). Similarly, in the down-regulated panel, proteins involved in the processes of cell adhesion (CTNNB1, FSCN1), Ca and/or actin binding (S100A2, S100A16, caldesmon/CALD1), glutathione metabolism (glutathione S-transferase GSTP1), as well as the epithelial cell marker and tumor suppressor 14–3–3 sigma (SFN)-a p53-regulated inhibitor of G2/M progression^46^, displayed the largest change.

The list of 352 proteins could be associated with all major cellular compartments (Fig. 9a). Associations with epithelium and various cancers were common, and matches to plasma, blood, milk, placenta and fetal brain cortex qualified among the top categories with 2–14-fold enrichment values and FDRs <6% (Supplemental Table S3). A large proportion of these proteins were cell surface/plasma membrane or extracellular space-related, displaying thus potential as therapeutic targets or diagnostic biomarkers. Notably, in recent years, the important role of exosomes in cell-cell communications and waste management has been recognized, as well as the many new opportunities that these extracellular vesicles offer for regenerative medicine applications^47^. Their biochemical cargo (proteins, lipids, mRNA, microRNA, non-coding RNA) has particular value to biomedical and clinical research, as the proteins are expected to be representative of the cellular source and status. A total of 132 proteins, illustrative of the biological processes that were described above, were associated with the exosome. Roughly ~60% of these proteins were shared with the plasma membrane/cell surface category, and ~20% of each category was identifiable in blood or plasma (Fig. 9b and c), a provoking result that elicits encouraging prospects for the development of tissue imaging and minimally invasive screening assays.

## Conclusions

All together, this study suggests that experimental results generated by cell cycle studies can be probed with public domain data to identify functionally-related protein panels with biomarker or drug target potential. Controlled biological interventions in cell cycle progression, followed by MS proteomic profiling and functional analysis, can reveal novel protein associations and evidence for previously unsuspected mechanisms that drive cancer cell proliferation. The use of prior knowledge can provide context and insight into data interpretation to inform future experimental design. The availability of protein panels reflective of the mechanistic aspects of cancer progression and metastasis will support the development of the most novel therapeutic approaches, e.g., immune response-based or PPI network targeted therapies. Translational efforts will benefit at all levels, from cancer detection and treatment, to monitoring therapeutic response and disease progression. The availability of large panels of putative biomarkers, as well as of the biological context in which such biomarkers manifest themselves, can inform the design of clinical trials, speed up and enhance the quality of the disease classification and staging process, refine the scope of targeted therapies, and ultimately advance the capabilities of precision medicine and companion diagnostics applications.

## Methods

### Materials

The MCF7, SKBR3 and MCF10 cell lines, as well as Eagle’s minimum essential medium (EMEM), trypsin (0.25%)/EDTA (0.53 mM), and phosphate-buffered saline (PBS) were purchased from ATCC (Manassas, VA). Phenol-red free Dulbecco’s modified Eagle’s (DMEM)/F-12 (1:1) and horse serum (HS) were from Invitrogen (Carlsbad, CA), McCoy 5 A was from Life Technologies (Carlsbad, CA), and human epidermal growth factor (hEGF) from PeproTech (Rocky Hill, NJ). Fetal bovine serum (FBS) for MCF7 and MCF10 cultures was from ATCC, and for SKBR3 from Gemini Bio-Products (West Sacramento, CA). Charcoal/dextran treated FBS was purchased from Hyclone (Logan, UT). Other cell culture or processing reagents such as 17-β estradiol (E2), hydrocortisone, cholera toxin, L-glutamine, bovine insulin, protease inhibitor cocktail (P8340), Na_3_VO_4_, NaF, urea, dithiothreitol (DTT), acetic acid, trifluoroacetic acid (TFA), ammonium bicarbonate (NH_4_HCO_3_) and all bovine protein standards (hemoglobin α/β, α-casein, β-casein), and the Cell Lytic™ NuCLEAR™ extraction kit were purchased from Sigma (St. Louis, MO). Trypsin, sequencing grade, was from Promega (Madison, WI). Sample cleanup SPEC-PTC18 and SPEC-PTSCX pipette tips were from Agilent. Methanol and acetonitrile, HPLC grade, were purchased from Fisher Scientific (Fair Lawn, NJ). Deionized water was produced in-house with a MilliQ Ultrapure water system (Millipore, Bedford, MA).

### Cell culture

The cells were cultured in an incubator, at 37 °C and 5% CO_2_, per manufacturer’s protocol. The cells were arrested for 48 h in serum-free medium, and released in culture medium supplemented with growth factors or hormones^48,49^ until a maximum proportion of S-fraction cells was obtained (24 h for MCF/MCF10 and 36 h for SKBR3). The culture medium for MCF7 was EMEM with FBS (10%) and bovine insulin (10 μg/mL); for MCF10 was DMEM/F12 (1:1) with horse serum (5%), EGF (20 ng/mL), hydrocortisone (0.5 μg/mL), cholera toxin (0.1 μg/mL) and insulin (10 μg/mL); and, for SKBR3 was McCoy 5 A with FBS (10%). The arrest medium for MCF7 was DMEM (phenol red-free) with L-glutamine (4 mM), for MCF10 was DMEM/F12 (1:1), and for SKBR3 was McCoy 5 A. The release medium for MCF7 was DMEM (phenol red-free) with L-glutamine (4 mM), FBS (10%, charcoal stripped), E2 (1 nM) and bovine insulin (1 μg/mL); for MCF10 was DMEM/F12 (1:1) with horse serum (10%), EGF (20 ng/mL), hydrocortisone (0.5 μg/mL), cholera toxin (0.1 μg/mL) and insulin (10 μg/mL); and for SKBR3 was McCoy 5 A with FBS (10%) and EGF (150 ng/mL). The cells were detached with 0.25% trypsin (0.25%)/EDTA (0.53 mM), harvested, and stored at −80 °C until further processing. FACS results (Beckman Coulter EPICS XL-MCL, Brea, CA) with propidium iodide stain included: (a) G1-arrested cells: 78–82% G1, 9–11 S %, 5–11% G2/M (MCF7); 91–92% G1, 6% S, 4–5% G2/M (MCF10); 72–80% G1, 13–17% S, 7–14% G2/M (SKBR3); and (b) S-released cells: 25–35% G1, 58–62% S, 8–12% G2/M (MCF7); 52–62% G1, 30–38% S, 9–16% G2/M (MCF10); 45–51% G1, 30–44% S, 13–18% G2/M (SKBR3). Three biological replicates, i.e., a new cell batch from the frozen stock, were cultured for each experiment.

### Protein extract preparation

The cells were separated into cytoplasmic and nuclear fractions with the Cell Lytic™ NuCLEAR™ kit supplemented with DTT (1 mM), protease inhibitor cocktail (1% of total lysate volume) and phosphatase inhibitors (Na_3_VO_4_ and NaF, 1 mM each). The concentration of proteins in the cell extracts was determined with the Bradford assay (SmartSpec Plus spectrophotometer, Bio-Rad, Hercules, CA). Bovine standards were added to the protein extracts at this stage (10 µL of 5 µM standards per 500 µg extract). The protein extracts were denatured/reduced with urea (8 M)/DTT (4.5 mM) for 1 hour at 55–60 °C, and digested with trypsin at a ratio of (30–50):1 (substrate:enzyme) for 24 hours at 37 °C. Salts and detergents were removed with SPEC-PTC18 and SPEC-SCX cartridges. The samples were prepared for LC-MS/MS analysis at 2 μg/μL in CH_3_CN/H_2_O/TFA (95–98):(2–5):0.01 v/v^50,51^.

### Protein extract analysis by LC-MS

LC-MS/MS analysis was performed with a micro-LC 1100 system (Agilent Technologies, Palo Alto, CA) coupled to a linear trap quadrupole (LTQ) mass spectrometer (Thermo Electron Corporation, San Jose, CA) via an in-house built interface and electrospray ionization (ESI) source^50^. The LC-MS interface facilitated LC system operation at flow rates of 10 µL/min, split-flow of ~180 nL/min for enabling nano-LC separations, and on-column/no-split injections. The nano-separation columns were built in-house from fused silica capillaries (100 μm i.d. × 360 o.d. × 12 cm long) packed with 5 μm Zorbax SB-C18 particles (Agilent). The ESI emitter, operated at 2 kV, was prepared by inserting a fused silica capillary (20 μm i.d. × 90 μm o.d. × 10 mm long) into the nano-separation column. The LC mobile phases were prepared from H_2_O:CH_3_CN:TFA mixed in a ratio of 95:5:0.01 v/v and 20:80:0.01 v/v for mobile phases A and B, respectively.

The separation gradient was 200 min long, where the concentration of eluent B was raised to 10% after sample loading on the separation column, and then to 35%, 45%, 60%, and 100% over 135, 50, 13, and 1 min, respectively. MS/MS data acquisition was performed using a data-dependent acquisition strategy with zoom/MS^2^ scans acquired for the five most intense peaks from a preliminary MS survey scan. The data-dependent acquisition parameters were set for ±5 m/z zoom scan width, ±1.5 m/z exclusion mass width, dynamic exclusion at repeat count 1, repeat duration of 30 s, exclusion list size 200, and exclusion duration 60 s. The tandem MS collision induced dissociation parameters were set for normalized collision energy 35%, activation Q 0.25, activation time 30 ms, and isolation width 3 m/z. Five technical replicates were performed for each protein extract sample, a technical replicate being defined in this study as a new LC-MS/MS analysis of the same sample.

### MS data processing

Discoverer 1.4 (Thermo Electron, San Jose, CA) and a minimally redundant human protein database from UniProt (20,198 reviewed, non-redundant protein sequences, January 2015 download) were used to process the raw MS files. The database search parameters included: fully tryptic peptides only, maximum two missed tryptic cleavages, 500 Da first mass, 5000 Da last mass, 2 Da precursor ion tolerance, 1 Da fragment ion tolerance, b/y/a ion fragments included in the search, no posttranslational modifications, and peptide-level stringent FDR <1% and relaxed FDR <3%.

### Statistical analysis

Quantitative comparisons were performed based on spectral count data. The five LC-MS/MS technical replicates of each sample were combined into one file to increase the number of protein IDs per cell state and the reproducibility of their detection in biological replicates. The protein counts were normalized based on the average spectral count data of all cell states. One spectral count was added after normalization to each protein to compensate for missing values. Comparisons between cancerous (MCF7, SKBR3) and non-cancerous (MCF10) cell states were performed by using log_2_-transformed values of protein spectral counts, and applying a Student *t*-test to select individual proteins for the up/down-regulated panels (two-sample comparisons, two-tailed/unpaired test, n_1_ = 3, n_2_ = 3, α/2 = 0.1, assumption of normally distributed mean protein abundance values with equal variances for the population of cancerous and non-cancerous cells).

### Bioinformatics data analysis

Protein annotations, enrichment, and functional analysis were performed with DAVID 6.8, by using a *Homo sapiens* background, with the thresholds set to count 2 and EASE score 0.1. PPI networks were explored with STRING 8.3, having the interaction score confidence set to medium/high. Maximum five interaction sources (1^st^ shell) are shown in each figure (i.e., textmining, experiments, databases, co-expression, neighborhood, gene fusion, and co-occurrence). Color-coding of functionally-related clusters was performed manually. Heat maps were produced in Excel, based on log_2_(FC) in spectral counts comparisons between cancerous and non-cancerous cell states. Circos plots were generated with standard parameters provided by the developer^52^.

### Data availability

The data analyzed in this study are included in the Supplementary Information files. Raw files are available from the corresponding author per reasonable request.

## Electronic supplementary material

Supplementary Information

Supplementary Dataset 1: Biomarker protein lists

Supplementary Dataset 2: Up/down-regulated protein panels

Supplementary Dataset 3: DAVID annotations and classification

Supplementary Dataset 4: Proteins included in Circos plots
