# Supplementary material for: Cell Cycle Model System for Advancing Cancer Biomarker Research
Source: Sci Rep. 2017 Dec 21;7:17989. doi: 10.1038/s41598-017-17845-6 (PMC5740075; doi:10.1038/s41598-017-17845-6)

Cell Cycle Model System for Advancing Cancer Biomarker Research

[1,2]Iulia M. Lazar,* [3]Ina Hoeschele, [1]Juliana de Morais, [1]Milagros J. Tenga

[1]Department of Biological Sciences and [2]Carilion School of Medicine, Virginia Tech
1981 Kraft Drive, Blacksburg, VA 24061

[3]Department of Statistics and Biocomplexity Institute, Virginia Tech
1015e Science Circle, Blacksburg, VA 24061

*To whom correspondence should be addressed
Iulia M. Lazar
Department of Biological Sciences
Virginia Tech
1981 Kraft Drive, Blacksburg, VA 24061
Email: malazar@vt.edu

Figure S1. STRING diagrams of protein-protein interaction networks. (**a-c**) Cell-line level PPI networks for cancer marker proteins identified in the MCF7, SKBR3 and MCF10 cells; (**d-g**) Cell cycle PPI networks for cancer marker proteins identified in the G1N, G1C, SN and SC cell cycle stages and cellular fractions; (**h-i**) Differential expression PPI networks for the up- and down-regulated protein panels. The number of proteins included in each network is shown in each figure, and the three overarching hubs of networks are highlighted in color: DNA damage repair (red), signaling (yellow), and oxidative phosphorylation/metabolism (purple).

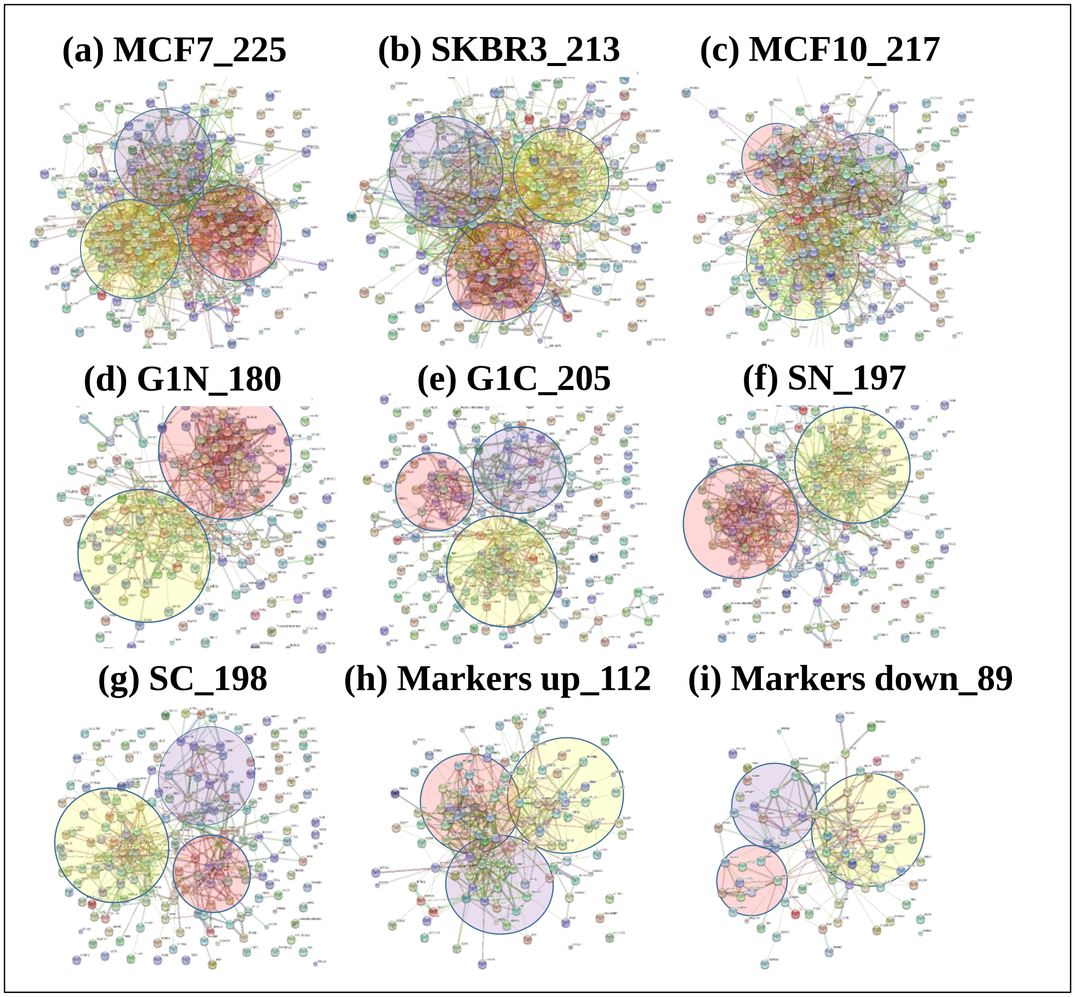

Supplement: Supplementary file 1 — Supplementary Information [file 41598_2017_17845_MOESM1_ESM.pdf]
